# Consensus for vaginal stenosis prevention in patients submitted to pelvic radiotherapy

**DOI:** 10.1371/journal.pone.0221054

**Published:** 2019-08-09

**Authors:** Sabrina Rosa de Lima Matos, Mariana Lucas Rocha Cunha, Sergio Podgaec, Eduardo Weltman, Ana Fernanda Yamazaki Centrone, Ana Carolina Cintra Nunes Mafra

**Affiliations:** 1 Radiation Oncology Department, Hospital Israelita Albert Einstein, São Paulo, São Paulo, Brazil; 2 Nursing School, Faculdade Israelita de Ciências da Saúde Albert Einstein, São Paulo, São Paulo, Brazil; 3 Women’s Health Department, Hospital Israelita Albert Einstein, São Paulo, São Paulo, Brazil; 4 Oncology Center, Hospital Israelita Albert Einstein, São Paulo, São Paulo, Brazil; 5 Hospital Israelita Albert Einstein, São Paulo, São Paulo, Brazil; University of South Alabama Mitchell Cancer Institute, UNITED STATES

## Abstract

**Objective:**

To propose a consensus for prevention of vaginal stenosis in patients submitted to pelvic radiotherapy.

**Method:**

In this methodological study, Delphi technique was applied for content validation on vaginal stenosis prevention. Data regarding content validation were collected from 32 specialists practicing in the oncology profession. The content validity index of items in the consensus was calculated based on the evaluations by the specialists.

**Results:**

In the first round, of the 38 items evaluated, 29 items reached a Content Validity Index (CVI-I) greater than 80%, and 9 items had a CVI lower than 80%. Of the items that did not obtain agreement, 2 items were excluded, and 7 were reformulated and included in the second round. In the second round, all 7 items obtained a CVI-I greater than 80%. The final instrument consisted of 29 items validated in the first round, plus 7 items reformulated and consolidated in the second round. The judges agreed that it is the responsibility of the health professionals to consult the patients undergoing radiation therapy in the area of sexuality to patients. The radiation oncologist should be the first professional to address this issue and the nurse oncologist in the follow-up consultation should pass the guidelines to the patients as comprehensively as possible. Patients should be informed about vaginal dilation, regardless of whether they are sexually active or have a partner. They should also be informed of when they can resume sexual activity. The procedure of vaginal dilation should be individualized. The prescribed vaginal dilators should be used with a lubricant for a duration of at least 5–10 minutes, 2–3 times a week, as per the need of each patient (sexual activity and/or clinical follow-up) for an indefinite time. Patients should seek medical help in case they experience pain, discomfort, or bleeding during dilation.

**Conclusion:**

The Brazilian version of the consensus for vaginal stenosis prevention in patients submitted to pelvic radiotherapy was validated with 36 items in 7 categories related to Responsibility; Target population; Rationale; Vaginal dilator; Content instructions; Information provision; and Patient support. In Brazil, the educational practices on vaginal dilation for patients submitted to radiotherapy partly revealed similar difficulties as identified in other studies as well as countries with reference to specific guidelines for the start and duration of vaginal dilation. The final consensus developed in this study could strengthen the guidelines for education of patients in Brazil and provide a future scope to establish a single and safe guideline.

## Introduction

According to the World Health Organization (WHO), cancer is a public health problem. Globally, breast cancer is the most commonly occurring cancer (25%) in women, while cervical cancer is the fourth most commonly occurring cancer (7.6%). In Brazil, cervical cancer is the third most frequent cancer with 16,370 new cases estimated to be diagnosed between 2018 and 2019, making it the second most frequent cause of death due to cancer in this period. [1, 2]

Pelvic radiotherapy is a common treatment for gynecological cancer. Nevertheless, it can result in long-term vaginal changes, such as decreased lubrication and vaginal stenosis, characterized by vaginal canal obstruction due to scar tissue formation. Vaginal stenosis can be identified by complaints such as difficulties in sexual intercourse as notified by the patient and those assessed by health professionals during gynecological examination, which may make the patient’s clinical treatment challenging. [3, 4, 5, 6]

Further, radiation causes damage to the vaginal epithelium, connective tissue, and small blood vessels, causing inflammation and local cell death leading to decreased local blood flow, tissue hypoxia, loss of elastin, collagen deposition, and hyalinization. These processes cause weakening of the vaginal mucosa, loss of lubrication, and scarring and fibrosis making the vagina shorter, less elastic, and dry as well as affecting the patients’ quality of life, especially regarding sexuality and self-esteem. [7, 8, 9, 10]

Interventions for vaginal stenosis prevention are based on limited scientific evidence suggesting the regular use of dilators to prevent or minimize adhesions, thereby separating the vaginal walls. Studies in Australia and the United Kingdom have evaluated the best practices in radiotherapy services regarding the time to start dilation, frequency of dilator use, time of each intervention (in minutes) and period (in months or years) that the use of the dilator should be maintained. [6, 9]

However, owing to the lack of a single strategy, there is a gap in the guidelines for vaginal dilator use in the prevention of vaginal stenosis after radiotherapy, especially in the Brazilian context, which may lead to inadequate guidelines or even the absence of suitable care for the patient. Even in the international context, there is no single strategy guiding prevention of this condition. It is important to note that the studies that address this problem agree on the need to provide education and support for patients submitted to pelvic radiotherapy. [7, 8]

Considering the impact of the occurrence of vaginal stenosis in women undergoing pelvic radiotherapy, as indicated by the literature, and the lack of a protocol of care in Brazil, we aimed to develop a national consensus on the prevention of vaginal stenosis in patients submitted to pelvic radiotherapy. The Brazilian consensus was adapted from the recent Dutch study on the use of dilators in the prevention of this condition in women undergoing radiotherapy [4].

## Methods

The project was approved by the Research Ethics Committee of the Albert Einstein Israelite Hospital (CAEE No. 62612616.2.0000.0071), in accordance with the norms and ethical precepts established in Resolution 466/2012, which deals with the Guidelines and Norms Regulating Research involving humans.

The study was carried out to validate the contents of the Dutch Consensus, [4] with the application of the Delphi Technique [11] and through the evaluation of these items by a committee of specialists in this field. The Dutch Consensus provides details on the information that should be offered to patients with gynecological cancer who undergo radiotherapy (RT) in support of the use of vaginal dilators and sexual rehabilitation. The consensus consists of 7 categories: “Responsibility,” “Target Population,” “Rationale,” “Vaginal Dilator,” “Content Instructions,” “Information Provision,” and “Patient Support.”

To facilitate the organization of the questionnaire, the Dutch consensual statements were presented in a numerical sequence with instructions for comments from the judges. The numerical sequence was freely chosen by the authors of this study (Table 1).

**Table 1:** Summary of the Dutch consensus-based recommendations described per category.

Category	Consensus
Responsibility	1. Health care providers should give patients simple sexological advice, such as how to cope with fear for sexual contact after treatment. 2. It is desirable to refer patients to a sexologist in case simple sexological advice does not suffice.
Target population	Patients should be informed about vaginal dilation in case they were: 3. Sexually active before treatment (independent of whether they have a partner). 4. Treated with RT for cervical or vaginal cancer. 5. Treated with vaginal brachytherapy in combination with external beam RT (or on individual indications).
Vaginal dilator	6. Health care providers should advise on which type of dilator should be used, but the patient ultimately decides. 7. The most often recommended type of dilator are commercially available plastic dilator sets. 8. Patients may use a vibrator if preferred. 9. The circumference of a dilator is important during usage.
Rationale	The rationale that health care providers use to prescribe vaginal dilation should contain that dilation: 10. Prevents the formation of vaginal adhesions. 11. Keeps the vagina accessible for any form of penetration in the future. 12. Also makes future vaginal examination (during follow-up appointments) more convenient. 13. Can be useful to help reduce fear for bodily changes and sexual activity. 14. Vaginal dilation should start preventively and not only in case of established adhesion.
Content instructions	15. Plastic cylinders, vibrators, dildos, and fingers should be inserted at least 1 to 3 minutes, 2 to 3 times a week, and during 9 to 12 months after treatment. 16. Vaseline tampons (tampons covered in vaseline) should be inserted overnight, 2 to 3 times a week, and during at least 9 to 12 months after treatment. 17. Lubricants should be advised together with vaginal dilators. 18. Gradually using a bigger cylinder circumference in time is important. 19. It is best to insert vaginal dilators as deep as possible, in a position determined by the patient herself, and to move the dilator around when inserted. 20. Patients should consult their health care provider in case of new complaints about pain or lasting loss of blood. 21. Whether or not the partner is actively involved should depend on the patients’ needs. 22. The frequency of use can be lowered in case the patient also has successful sexual intercourse. 23. Patients may start having sexual intercourse 2 to 4 weeks after treatment.
Information provision	24. The health care center decides which health care provider is responsible for informing patients about vaginal dilation. 25. The radiation oncologist should provide the first introduction before RT. 26. The oncology nurse should provide the more extensive information during the first follow-up appointment. 27. The health care provider should initiate information provision, at least face-to-face, even if the patient does not begin to talk about it. 28. The patients’ partners should be involved. 29. The availability of an informational brochure and Web site is desirable.
Patient support	30. The health care center decides which health care provider is responsible for supporting patients during sexual rehabilitation. 31. Monitoring vaginal dilator use should always take place during follow-up appointments. 32. The oncology nurse should provide psychological and practical patient support during sexual rehabilitation. 33. The health care provider should initiate providing patient support even if the patient does not take the initiative. 34. Extra consultations to support the patient should be possible. 35. Extra referral possibilities for patients with sexual problems and more training possibilities in assessing sexual complaints are desirable.

The Dutch consensus was modified by the authors of the present study by adding 3 extra items, in 2 categories, after which the contents were sent for evaluation by the judges. The extra items are shown in Table 2.

**Table 2:** Extra items inserted in the protocol for the prevention of vaginal stenosis in patients submitted to pelvic radiotherapy.

Category	Extra item
Target population	**Extra 1**: “Patients with vulvar or endometrial cancer and/or who were not sexually active prior to treatment should receive care tailored to their needs”.
	**Extra 2**: “Vaginal dilation may be recommended in individual situations for women who have received pelvic radiotherapy for colorectal and anal tumor”.
Content instructions	**Extra 3**: “Vaginal dilation should be initiated 2 to 4 weeks after completion of radiotherapy or when the vaginal mucosa has recovered (around 4 weeks)”.

Items 1 and 3, mentioned above, were addressed in the Dutch study [4]. However, according to published results, it was not possible to obtain the expected validation index for the consensus. The "Extra 2" item was proposed on the basis of the *International Guidelines* [12] of the United Kingdom, which suggests vaginal dilation in patients undergoing pelvic radiotherapy for colorectal and anal tumors. We found it necessary to address these items so that there was no gap in this aspect in to prevent the validation of the protocol. Therefore, the instrument sent to the judges consisted of 38 items subdivided into the same 7 original categories.

Before starting the translation process, the authors of the Dutch Consensus consented to the translation of the document to be used in Brazil.

The cross-cultural translation process was based on the "*Recommendations for the Cross-Cultural Adaptation of the DASH & Quick DASH Outcome Measures*". [13] It was carried out by two independent Brazilian translators, both English proficient, with one being a health professional. After this step, a combined version of the two translations was retranslated to the original (English) by another translator whose native language was English (Blinded back translation). This bilingual translator formally evaluated the equivalence between the back-translation and the language in the original instrument, thus concluding the final translation.

Before the instrument was sent for expert professional evaluation, a pilot (pre-test) assessment stage of the material submission tool was carried out, where a professional from each area of the target audience was selected to evaluate the level of comprehension of the items. The team was composed of a nurse, a radiation oncologist, an oncology gynecologist and a physiotherapist. The objective of this stage was to evaluate whether the terminology (the semantics) or the practical behaviors, were culturally acceptable after translating the items and cross-cultural adaptation. The task of these professionals in this stage was not to evaluate the content validity for each category item. It was also important to perform the usability test of the data collection platform and to verify possible difficulties that the experts might face (in the Google Forms case).

In this discussion, 10 items were adjusted, namely items 1, 2, 4, 7, 15, 22, 23, 28 and 32. In item 2, the Dutch consensus proposes to refer the patient, if necessary, to a sexologist, a professional who is not part of most health teams in Brazil. The suggestion was adapted to the Brazilian culture to include other professionals specialized in the subject.

Regarding item 7 on the type of dilators available for dilatation, the Dutch consensus suggested that the plastic dilator was the one most widely used. However, experts in the pre-test panel decided that it is important to add other types of dilators with different materials, such as silicone, to the list. Item 15 complements item 7, it was necessary to modify the beginning of the phrase referring to the type of dilator.

In items 1, 4, 22, 23, 28, and 32, the changes were related to terminology after cultural translation, for example, in item 1, the word “sexology” was replaced by “sexuality.”

Data were collected from August 2017 to February 2018 through individual forms sent to the specialists electronically through the Google Forms website. Selection of the sample of specialists was done using the *Snowball* technique, which is a non-probabilistic sampling technique that uses reference chains, making it useful for recruiting studying groups that are difficult to access. [14]

Table 3 presents the judges’ profile in the first round. Of the 32 judges that participated, 65.6% were women aged between 28 and 65 years (median = 37.5 years). As for the profession, 31.2% were nurses, 28.1% radiation oncologists, 25.0% gynecologists or oncological surgeons, and 15.6% were physiotherapists. Most of the professionals had more than seven years of experience in their respective fields (65.6%). The majority of judges (23; 71.9%) were from southeast region of Brazil.

**Table 3:** Profile of responding judges (n = 32).

Profile of judges	Descriptive measures
**Gender (%)**	
Female	21 (65.6)
Male	11 (34.4)
**Age**	
Median [IQI]	37.5 [35.0; 43.25]
**Profession (%)**	
Nurse	10 (31.2)
Physiotherapist	5 (15.6)
Gynecologist / Oncology Surgeon	8 (25.0)
Radiation Oncologist	9 (28.1)
**Time working in the previously described area**	**N (%)**
Between 1 and 3 years	5 (15.6)
Between 4 and 6 years	6 (18.8)
Between 7 and 10 years	8 (25.0)
More than 10 years	13 (40.6)
**Region of Brazil**	
Southeast	23 (71.9)
South	4 (12.5)
North	3 (9.4)
Northeast	2 (6.3)

IQI: Interquartile Interval (1^st^; 3^rd^ quartiles).

The Content Validity Index of Items (CVI-I) [15] was calculated as the sum of the evaluations, "I agree" or "I totally agree" on the item divided by the sum of all evaluations on the item. For the final version of the instrument, the Content Validity Indices of Axis (CVI-A) were calculated as the mean of the CVI-I in each axis (category), and the Content Validity Index of the Questionnaire (CVI-Q) as the mean of all CVI-I of the instrument.

Two rounds of the Delphi Method were required for the CVI-I to reach the established 80% criterion.

### Statistical analyses

At the end of each round, the CVI-I, and the percentages and absolute frequencies of responses were described.

In each round and in the finalization of the instrument, inter-rater agreement coefficients were calculated based on the judges’ Likert scale responses through agreement coefficient AC2, [16] with ordinal weights appropriate for the scenario in which the answers follow an ordinal scale (as in the case of the Likert scale), and if there are more than two evaluators. The results are presented as estimated coefficients, 95% confidence intervals, and *p* values for hypothesis testing.

The analyses were conducted with the program R (R Core Team, 2017), version 3.4.1 [17]. The level of significance was 5%.

## Results

Validation was performed in two rounds as presented below.

### First round

In the first round, 38 items were evaluated, and from these evaluations, the agreement coefficient AC2 among 32 judges was 0.75 with a 95% confidence interval between 0.69 and 0.81 (p-value <0.001).

For cases where a judge replied "Disagree" or "I totally disagree", he/she was still asked if the item should be kept with adjustments or if it should be deleted. Of these items, 26 were evaluated in this aspect by at least two judges. The agreement coefficient AC2 between them was 0.63 with a 95% confidence interval between 0.40 and 0.86 (p-value <0.001).

**Fig 1:**
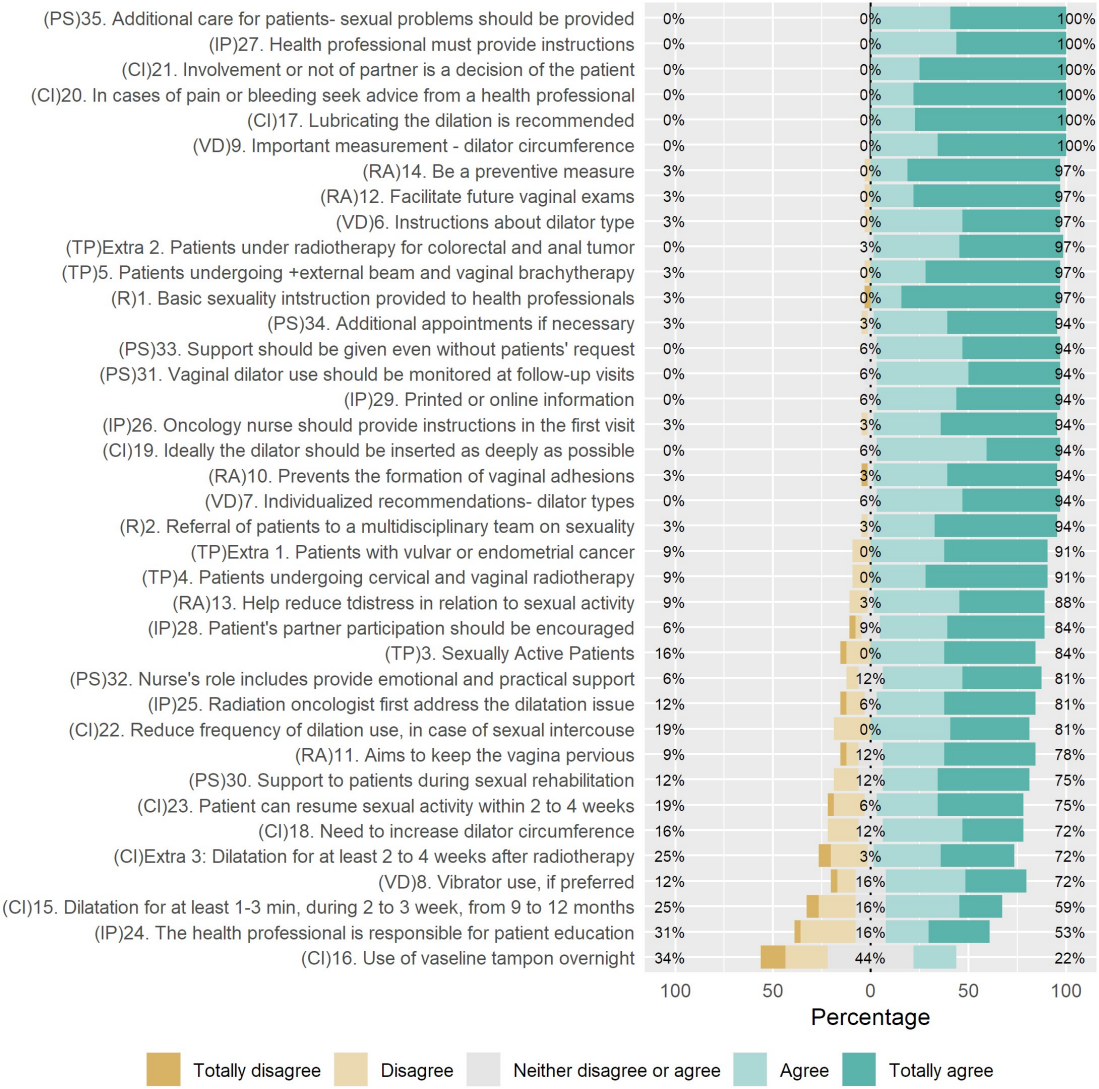
Distribution of answers provided by judges to the items in the first round. N = 32 judges. Responsibility (R), target population (TP), vaginal dilator (VD), rationale (RA), content instructions (CI), information provision (IP), and patient support (PS).

Fig 1 shows the distribution of the judges’ responses according to the Likert Scale. Among the 7 categories presented, 5 of them (Vaginal dilator, Rationale, Content instructions, Information provision, and Patient support), had 9 items with CVI-I less than 80% the items were: 08, extra 3, 11, 15, 16, 18, 23, 24, and 30. The highest degree of disagreement was regarding the category "Content instructions", which had 5 items, with CVI-I lower than 80%, and among them, “item 16” presented the lowest CVI-I (22%).

### Second round

The same judges were invited to the second round, but 5 professionals (2 nurses, 2 gynecologist/oncological surgeons, and a physiotherapist) did not participate in the second round. Twenty-seven judges (84.4%) completed the second round, of which 29.6% were nurses (8), 14.8% were physiotherapists (4), 22.2% were gynecologists/oncological surgeons (6), and 33.3% radiation oncologists (9).

From the first round, the items "extra 3" (referring to the time to start the dilation process) and "16" (on the use of vaseline tampon) were excluded from the instrument. For the time of onset of dilatation (extra item 3 obtained 72% agreement), the judges’ suggestions varied so that it was not possible to reformulate the item. The use of petroleum jelly tampons (vaseline) obtained a very low level under agreement of the judges (22% of concordance), which made it impossible to reformulate the item. Items 8, 11, 15, 18, 23, 24, and 30 that obtained CVI-I below 80% were rewritten (Table 4). After the changes were made (second round version), those same items were returned to the judges for the second round evaluation.

**Table 4:** Comparison between items in the 1^st^ round and the modified items of the 2^nd^ round.

Category	1st Round Version	2nd Round Version
Vaginal dilator	8. “Patients may use a vibrator if preferred”.	8. Patients may use a vibrator if preferred, provided the vibrator has the appropriate shape for the size of the vagina. "
Rationale	11. “Keeps the vagina accessible for any form of penetration in the future”.	11. “It aims to keep the vagina accessible for any form of penetration in the future, such as sexual intercourse and gynecological examinations.”
Content instructions	15.” The prescribed vaginal dilators should be inserted for a duration of at least 1–3 minutes, 2–3 times a week, and for 9–12 months after treatment”.	15. The prescribed vaginal dilators should be inserted for a duration of at least 5–10 minutes, 2–3 times a week, according to the need of each patient (sexual activity and/or clinical follow-up) for an indefinite time."
	18. Gradually using a bigger cylinder circumference in time is important.	18. “Over time the patient who had begun dilation with a vaginal dilator of a smaller diameter than the diameter of the vagina before treatment, should gradually use dilators with bigger circumferences until reaching a comfortable diameter and a suitable size of the vaginal canal.”
	23. “ Patients may resume sexual intercourse 2–4 weeks after the treatment if they feel ready”.	23. "Patients may resume sexual activity when they feel ready, which ideally should occur 2–4 weeks after treatment, when the vaginal mucosa is recovered.”
Information provision	24. “The health care center decides which health care provider is responsible for informing patients about vaginal dilation”.	24. “The health care institution should decide which professional of the multidisciplinary health team will be responsible to provide consultation on vaginal dilation.”
Patient Support	30. “The health care center decides which health care provider is responsible for supporting patients during sexual rehabilitation”.	30. “The health care institution should decide which professional of the multidisciplinary health team will be responsible for providing support to patients during the sexual rehabilitation process.”

For the seven reformulated items, the concordance coefficient AC2 among 27 judges was 0.81 with a 95% confidence interval between 0.74 and 0.87 (p-value <0.001).

**Fig 2:**
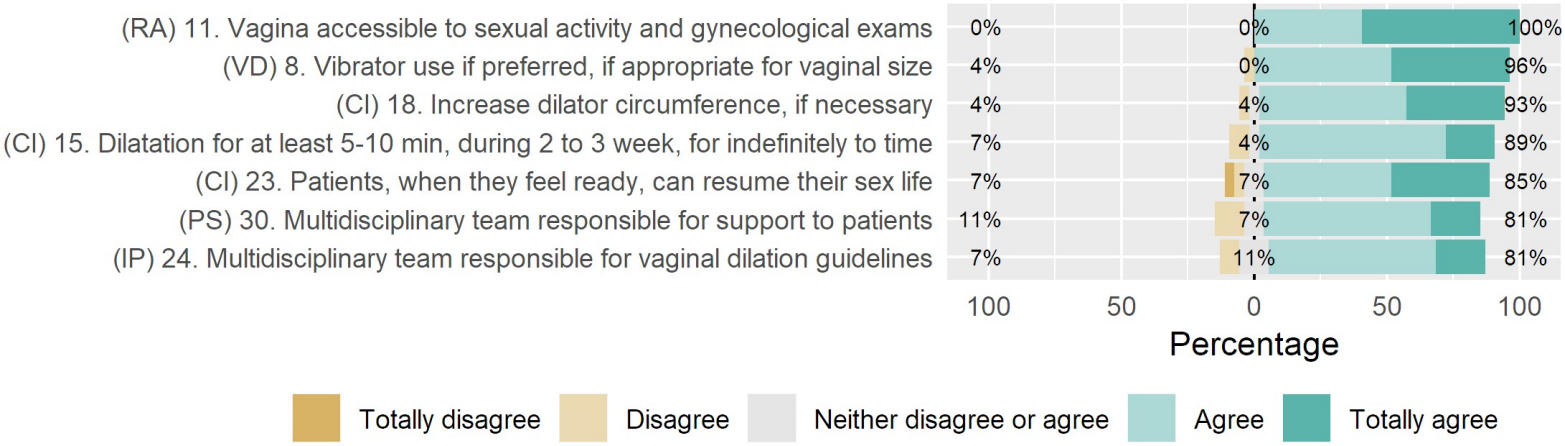
Distribution of judges’ answers to the reformulated items in the second round. N = 27 judges. Responsibility (R), target population (TP), vaginal dilator (VD), rationale (RA), content instructions (CI), information provision (IP), and patient support (PS).

Fig 2 shows the distribution of the judges’ answers to the items in the second round of which item 11 reached 100% agreement.

The final instrument consisted of 29 items validated in the first round, plus 7 items reformulated and consolidated in the second round, totaling 36 items.

When considering the last evaluations of these items, the agreement AC2 among judges was 0.82 with a 95% confidence interval between 0.78 and 0.85 (p-value <0.001).

The final CVI-Q of the instrument was 0.92. Table 5 presents the CVI-A for each evaluated category, where it is noted that CVI-A ranged from 0.89, in the category *Information provision* up to 0.97 in the *Vaginal dilator* category.

**Table 5:** Value of CVI-A according to the category assessed.

*Category*	*Number of items*	*CVI-A*
Responsibility	2	0.95
Target population	5	0.92
Vaginal dilator	4	0.97
Rationale	5	0.95
Content instructions	8	0.93
Information provision	6	0.89
Patient support	6	0.91

Table 6 (Brazilian Portuguese version) and S1 Table (English version) present the final consensus of instructions for the prevention of vaginal stenosis in patients submitted to pelvic radiotherapy.

**Table 6:** Final consensus for vaginal stenosis prevention in patients submitted to pelvic radiotherapy—Brazilian Portuguese version.

*Categoria*	*Consenso Final*
Responsabilidade	† Os profissionais da área da saúde devem dar orientações básicas na área de sexualidade, por exemplo, como a paciente pode lidar com o medo de ter relação sexual após o tratamento. † Caso estas orientações sobre sexualidade não sejam suficientes, o ideal é encaminhar as pacientes a um psicólogo e/ou a outros profissionais da equipe multidisciplinar especializada na área de sexualidade.
População-alvo	As pacientes que devem ser informadas sobre a dilatação vaginal são aquelas: † Sexualmente ativas antes do tratamento (independente de terem ou não um parceiro). † Submetidas ao tratamento com radioterapia para câncer de colo do útero ou vaginal. † Submetidas ao tratamento com braquiterapia vaginal em combinação com radioterapia de feixe externo (ou em indicações individuais). † Com câncer de vulva ou endométrio e/ou que não eram sexualmente ativas antes do tratamento, devendo receber cuidados adaptados às suas necessidades. † Submetidas à radioterapia pélvica por tumor colorretal e anal, em situações individualizadas.
Dilatador vaginal	† Os profissionais da área da saúde devem orientar suas pacientes em relação ao tipo de dilatador que deverão usar, deixando claro que a decisão final caiba a elas. † A recomendação quanto ao tipo de dilatador vaginal a ser usado deve ser individualizada, de acordo com as seguintes opções: prótese peniana, dilatadores de plástico, dilatadores de silicone e/ou de outro material adequado para a região vaginal. ‡ Se preferirem, as pacientes podem usar um vibrador, desde que tenha um formato adequado ao tamanho da vagina. † Durante o uso, a circunferência do dilatador é uma característica importante.
Justificativa	A justificativa que os profissionais de saúde devem utilizar para prescrever a dilatação vaginal deve conter que a dilatação: † Previne a formação de aderências vaginais. ‡ Visa manter a vagina acessível à diferentes formas de penetração futura, tais como atividade sexual e exames ginecológicos. † Facilita a realização de futuros exames vaginais (realizados durante as consultas de seguimento). † Pode ajudar a diminuir a angústia da paciente tanto em relação às mudanças em seu corpo como em relação à atividade sexual. † Deve ser iniciada como uma medida preventiva, ao invés de corretiva, e não somente após o surgimento de aderências.
Conteúdo das orientações	‡ Os dilatadores vaginais indicados devem permanecer inseridos por pelo menos 5 a 10 minutos, duas a três vezes por semana, por tempo indeterminado, de acordo com necessidade de cada paciente (atividade sexual e/ou seguimento clínico). † Recomenda-se o uso de lubrificantes durante o uso de dilatadores vaginais. ‡ É importante que, com o tempo, a paciente que iniciou a dilatação com um dilatador vaginal de circunferência menor (circunferência menor comparada ao diâmetro da vagina pré-tratamento), passe gradualmente a usar dilatadores com circunferências cada vez maiores, até atingir um diâmetro confortável e o canal vaginal pérvio. † Na posição escolhida pela própria paciente, ela deve idealmente inserir o dilatador o mais profundamente possível e movimentá-lo após sua inserção. † Caso comecem a sentir dores ou apresentem sangramento duradouro, as pacientes devem consultar os profissionais de saúde que a atendem. † Ter ou não o envolvimento ativo do parceiro depende das necessidades e escolhas de cada paciente. † Caso a paciente tenha relações sexuais bem-sucedidas com penetração vaginal completa, ela pode diminuir a frequência do uso dos dilatadores. ‡ As pacientes, quando se sentirem aptas, podem retomar a sua vida sexual. O ideal é que ocorra quando a mucosa vaginal estiver recuperada, podendo ser entre 2 e 4 semanas após o tratamento.
Provisão de informação	‡ A instituição de saúde deverá decidir quem serão os profissionais da equipe multidisciplinar de saúde responsável por passar as orientações relativas à dilatação vaginal. † O médico rádio-oncologista deve ser o primeiro a abordar a questão com a paciente, sendo que deve fazê-lo antes do início da radioterapia. † Durante a primeira consulta de seguimento, a enfermeira oncologista deve passar as orientações da forma mais completa possível. † O profissional de saúde deve passar as orientações de forma presencial, mesmo que a paciente não toque no assunto. † A participação do parceiro da paciente deve ser encorajada. † É desejável disponibilizar informação impressa ou *on-line*.
Apoio à paciente	‡ A instituição de saúde deverá decidir quem serão os profissionais da equipe multidisciplinar de saúde responsável em oferecer apoio às pacientes durante o processo de reabilitação sexual. † O uso do dilatador vaginal deve ser monitorado em todas as consultas de seguimento. † A enfermeira oncologista deve prestar apoio emocional e prático à paciente ao longo de todo o processo de reabilitação sexual. † O profissional da saúde deve oferecer apoio à paciente mesmo que ela não tome a iniciativa. † Se necessário, é preciso que consultas adicionais sejam marcadas. † É desejável que as pacientes com problemas sexuais possam ser encaminhadas a outros tipos de atendimento e que outras oportunidades de aprendizado lhes sejam oferecidas para que aperfeiçoem sua capacidade de auto avaliação de queixas de natureza sexual.

^**†**^ Itens fechados na primeira rodada

^**‡**^ Itens fechado na segunda rodada

## Discussion

In our study, among the judges with four selected professions, the highest number of judges were nurses (31.2%), which may be justified by the role that this profession occupies in the care and guidance of patients who have undergone radiotherapy treatment. The judges had more than 10 years of experience in the field (40.6%). A similar trend was observed in other countries where nurses were accountable for providing educational information to prevent vaginal stenosis. [6, 9]

In the first round, of the 38 items evaluated, 29 items reached CVI-I> 80%; 2 were excluded, 7 items did not reach the required level of agreement and were adjusted and sent to the second round.

In the "Responsibility" category, two items reached a CVI-I> 90%, that is, the judges agreed on including the importance of giving basic instructions to the patients regarding sexuality and advising them about needing to consult other specialists on this subject. Previous studies have also emphasized the importance of discussing sexual issues with gynecological cancer patients, since they are subject to prejudice and suffering due to imposition of societal values and beliefs. [18]

In the “Target population” category, agreement was reached with CVI-I >80% on giving instructions to patients who received pelvic radiotherapy for gynecological tumors and, in individual situations, to women who received pelvic radiotherapy for colorectal and anal tumors. In the literature, vaginal stenosis is best described in patients who undergo pelvic radiotherapy for gynecological tumors, especially in patients with uterine cervix tumors. In one study, vaginal shortening was observed after brachytherapy with a higher incidence in patients with cervical cancer compared to patients with endometrial cancer. [19]

Vaginal stenosis in patients who undergo pelvic radiotherapy for colorectal and anal cancer is still not well described in the literature. A study that aimed to evaluate the incidence of vaginal stenosis in women with anal cancer treated with radiotherapy simultaneously with definitive chemotherapy indicated an expected toxicity, in a mean time of 13 months after completion of radiotherapy. [20] This reinforces the importance of new studies in this area, since it is still a restricted field and is scarcely addressed.

Although not investigated in this study, it seems that there is no consensus regarding the minimum radiation dose that causes vaginal toxicity, hence suggesting that vaginal dilation should only be made available to women receiving pelvic RT as determined by volume of treatment and dose in the vagina, as opposed to only the primary diagnosis of gynecological malignancy. These findings corroborate with a previous study. [6, 12]

In the category “Vaginal dilator”, it was defined in item 7 that the recommendation will be on a case-by-case basis, be it penile prosthesis, plastic or silicone dilators, and/or other suitable material for the vaginal region. This item was already changed in the pre-test, because in the Dutch Consensus, the set of dilators indicated were of rigid plastic. However, in the discussion with experts, it was deemed necessary to insert other types of dilators (with different types of materials), corroborating with what had already been pointed out as one of the limitations in the validation of the Dutch Consensus. [4]

This validation did not reach the agreement level (72%) in relation to the time/moment recommended for the beginning of dilation, that is the proposal was to initiate dilation 2 to 4 weeks after radiotherapy completion or when the vaginal mucosa had recovered. The suggestions varied from initiating the dilation during radiotherapy sessions to right after the end of radiotherapy, which made it impossible to obtain consensus and to send the item for analysis in the second round.

Corroborating this research, other studies have revealed difficulties in validating this orientation. In the Dutch Consensus, the level of agreement was even lower than that of the present study (67%). A study in Australia, found no agreement on the optimal time to start dilation, but most responded with around 4 weeks after radiotherapy was terminated. In a systematic review, it was concluded that there were no evidence-based supporting instructions of starting dilation in the acute toxicity phase of radiotherapy (during or shortly after radiotherapy). [4, 12, 21]

A recent study in Brazil, which aimed to determine the incidence of vaginal stenosis shortly after completion of pelvic radiotherapy for uterine cervix tumors and to evaluate the factors associated with the occurrence of this event, measured the length and diameter of the vagina using acrylic graduated cylinders of 1 to 20 cm in length and cylinders of 30 to 40 mm in diameter prior to the start of radiotherapy and shortly after completion. In this study, 10.8% of the patients had reductions in vaginal diameter and 65.7% in vaginal length shortly after radiotherapy. [22]

There are issues that must be considered in relation to the above-mentioned study since immediately after end of radiotherapy, there is local mucosal inflammation that may interfere with this evaluation. Thus, a long-term follow-up is necessary to verify significant differences and to be able to define what would be the best time to start vaginal dilation. It should be emphasized that the start of dilation, during or shortly after radiotherapy, may discourage patients from undergoing vaginal dilation. This item is fundamental in vaginal stenosis prevention process and the non-consolidation of judges’ opinions, both internationally and nationally, shows the importance of developing new studies that may bring more evidence to the professional’s decision making.

Item 16 that refers to the use of petroleum jelly tampons (vaseline tampons) had one CVI that fell short from the others after analysis of the judges’ justification, and for lack of scientific evidence for this indication, the item was considered inapt for the evaluation in the second round.

Regarding the time and frequency of dilator use, an insertion time of 5 to 10 minutes with a frequency of 2 to 3 times a week were defined in the present study, which are identical to practices defined in studies in the United Kingdom.[6,12] Indefinite time for dilator use has also been suggested in some studies [6, 9, 12]. Still, there is no definite consensus as to how long after radiotherapy can vaginal stenosis occur leading to the judges’ suggestion that dilation time can be defined based on each patient’s sexual activity and clinical follow-up.

Individual follow-up of patients is very important since there is no standard rule or recommendation for the duration of vaginal dilation for all patients, and it needs to be based on each patient’s sexual activity and in their clinical follow-up.

A healthcare professional must know how to "listen to, feel, discriminate, recommend, and prescribe or refer" the issues bothering the patient after treatment. [23] The study reinforced that the nurse, at the first follow-up visit, should provide guidelines as thoroughly as possible about dilation.

In other countries, there is a well-established practice that the nurse is responsible for giving instructions for dilation and support during the process. In one study, nurses received training and followed instructions on dilation and assisted these patients during each dilation session (monthly) and assessed their sexual function at 1, 6, and 12 months. The rate of adherence of the patients to the use of dilators was 88%. [24] On the other hand, in a similar study, it was identified that at 6 months (4 months after the dilation instructions), only 31% of the participants had conducted dilation at least twice a week. Patients also reported that the nurses’ support provided safety and motivated them to begin dilation. Having a specific nurse available for follow-up consultation was important to talk comfortably about the patient’s personal situation and sexual functioning. [25]

Among other issues, this research is relevant because it supports the importance of specific training for nurses, focusing on the dilation instructions based on the validated consensus considering the needs of each patient. This monitoring intervention can assist in the commitment and safety of radiotherapy patients. It is worth emphasizing the role of the nurse as an educator, since s/he is the professional who assists the patient in all the cancer treatment stages.

## Conclusion

This study allowed the validation of 36 items of the Brazilian version of a consensus for vaginal stenosis prevention in patients submitted to pelvic radiotherapy. The results indicated that the consensus meets most of the patient instruction needs and can be characterized as a guideline for health education that should be promoted by the professional. This consensus can be considered a reliable instrument for the education of patients in this framework.

The Brazilian consensus regarding specific instructions for the duration and start time of vaginal dilation revealed difficulties similar to international studies. It is important to emphasize the significance of new research in this area, to have scientific evidence to support instructions in these aspects. The training of nurses, or other professionals responsible for patient education, to establish a single and safe guideline (as mentioned in the Dutch study), is in line with the future scope of research.

We believe that the accomplishment of this study advanced the knowledge regarding a fundamental theme in oncology. Thus, the final consensus developed here could strengthen the guidelines for education of patients in Brazil and in countries with similarly diagnosed cases while considering the particularities of each treatment center and patient.

## Supporting information

S1 Table Final consensus for vaginal stenosis prevention in patients submitted to pelvic radiotherapy—English version. (DOCX)
