# Supplementary material for: Consensus for vaginal stenosis prevention in patients submitted to pelvic radiotherapy
Source: PLoS One. 2019 Aug 9;14(8):e0221054. doi: 10.1371/journal.pone.0221054 (PMC6688793; doi:10.1371/journal.pone.0221054)
Supplement: S1 Table — (DOCX) [file pone.0221054.s001.docx]

**S1** **Table -** Final consensus for vaginal stenosis prevention in patients submitted to pelvic radiotherapy – English version.

| **Category** | | **Consensus** |
| --- | --- | --- |
| Responsibility | | **†** Health professionals should give basic instructions on the topic of sexuality, for example, how the patient can cope with the fear of having sexual intercourse after treatment.  **†** If these instructions on sexuality are not enough, the ideal option is to refer patients to a psychologist and/or other professionals of the multidisciplinary team specialized in the area of sexuality. |
| Target Population | | The patients who should be informed about vaginal dilation are those:  **†** Who were sexually active before treatment (regardless of whether they have a partner).  **†** Who submitted to treatment with radiotherapy for cervical or vaginal cancer.  **†** Who submitted to treatment with vaginal brachytherapy in combination with external beam radiotherapy (or in individual indications).  **†** With vulvar or endometrial cancer and/or who were not sexually active prior to treatment, and who are receiving care tailored to their needs.  **†** submitted to pelvic radiotherapy for colorectal and anal tumors in individualized situations. |
| Vaginal dilator | | **†** Health professionals should instruct their patients on the type of dilator they should use, making it clear that the final decision is up to them.  **†** The recommendation for the type of vaginal dilator to be used should be individualized according to the following options: penile prosthesis, plastic dilators, silicone dilators and/or other material suitable for the vaginal region.  **‡** If they prefer, patients can use a vibrator, if it is shaped appropriate to the size of the vagina.  **†** During use, the circumference of the dilator is an important feature. |
| Rationale | | The justification for health professionals prescribing vaginal dilation should be that the dilation:  **†** Prevents the formation of vaginal adhesions.  **‡** Aims to keep the vagina accessible to different forms of future penetration, such as sexual activity and gynecological exams.  **†** Facilitates future vaginal exams (performed during follow-up visits).  **†** Can help reduce the patient's distress in terms of changes in her body and in relation to sexual activity.  **†** Should be initiated as a preventive measure, rather than corrective, and not only after the appearance of adhesions. |
| Content instructions | **‡** The indicated vaginal dilators should remain inserted for at least 5 to 10 minutes, two to three times a week, for an indefinite period, according to each patient's need (sexual activity and/or clinical follow-up).  **†** It is recommended to use lubricants during the use of vaginal dilators.  **‡** It is important that over time the patient who has begun using a vaginal dilator of smaller circumference (smaller circumference compared to the diameter of the pre-treatment vagina), gradually use dilators with increasing circumferences until reaching a comfortable diameter and patent vaginal canal.  **†** In the position chosen by the patient herself, she should ideally insert the dilator as deeply as possible and move it after insertion.  **†** If they begin to feel pain or have lasting bleeding, the patients should consult their health professionals.  **†** Having the patient’s partner’s active involvement or otherwise depends on the needs and choices of each patient.  **†** If the patient has successful intercourse with complete vaginal penetration, she may decrease the frequency of dilator use.  **‡** Patients, when they feel fit, can resume their sex life. The ideal is that it occurs when the vaginal mucosa has recovered, which can be between 2 and 4 weeks after treatment. | |
| Information provision | **‡** The health institution should decide who will be the professionals of the multidisciplinary health team responsible for conveying the instructions related to vaginal dilation.  **†** The radiation oncologist should be the first to address the issue with the patient and should do so before radiotherapy begins.  **†** During the first follow-up visit, the oncologist nurse should follow the instructions as thoroughly as possible.  **†** The health professional must convey the instructions in person, even if the patient does not touch the subject.  **†** The participation of the patient's partner should be encouraged.  **†** It is desirable to provide printed or online information. | |
| Patient support | **‡** The health institution should decide who will be the professionals of the multidisciplinary health team responsible for offering support to patients during the process of sexual rehabilitation.  **†** The use of the vaginal dilator should be monitored at all follow-up visits.  **†** The nurse oncologist should provide emotional and practical support to the patient throughout the entire process of sexual rehabilitation.  **†** The health professional should offer support to the patient even if she does not take the initiative.  **†** If necessary, additional appointments must be made.  **†** It is desirable that patients with sexual problems should be referred to other types of care and that other learning opportunities should be offered to them to improve their ability to self-assess sexual complaints. | |

**†** *Consensus reached in the first round*

**‡** *Consensus reached in the second round*
